# Self-assembled peptide-paclitaxel nanoparticles for enhancing therapeutic efficacy in colorectal cancer

**DOI:** 10.3389/fbioe.2022.938662

**Published:** 2022-09-28

**Authors:** Lidan Hou, Ting Zhong, Peng Cheng, Bohan Long, Leilei Shi, Xiangjun Meng, Han Yao

**Affiliations:** ^1^ Department of Gastroenterology, Shanghai Ninth People’s Hospital, Shanghai Jiao Tong University School of Medicine, Shanghai, China; ^2^ The Digestive Disease Research and Clinical Transformation Center, Shanghai Jiao Tong University, Shanghai, China; ^3^ Shanghai Key Laboratory of Gut Microecology and Associated Diseases, Shanghai, China; ^4^ Department of Gastroenterology, Hainan West Central Hospital, Hainan, China; ^5^ The Eighth Affiliated Hospital, Sun Yat-sen University, Shenzhen, China

**Keywords:** peptide, self-assembled, targeted therapy, nanoparticles, colorectal cancer

## Abstract

Chemotherapy is one of the main treatments for colorectal cancer, but systemic toxicity severely limits its clinical use. Packaging hydrophobic chemotherapeutic drugs in targeted nanoparticles greatly improve their efficacy and reduce side effects. We previously identified a novel colorectal cancer specific binding peptide P-LPK (LPKTVSSDMSLN) from phage display peptide library. Here we designed a self-assembled paclitaxel (PTX)-loaded nanoparticle (LPK-PTX NPs). LPK-PTX NPs displayed a superior intracellular internalization and improved tumor cytotoxicity *in vitro*. Cy5.5-labeled LPK-PTX NPs showed much higher tumor accumulation in colorectal cancer-bearing mice. Furthermore, LPK-PTX NPs exhibit enhanced antitumor activity and decreased systemic toxicity in colorectal cancer patient-derived xenografts (PDX) model. The excellent *in vitro* and *in vivo* antitumor efficacy proves the improved targeting drug delivery, suggesting that peptide P-LPK has potential to provide a novel approach for enhanced drug delivery with negligible systemic toxicity.

## Introduction

Chemotherapy is a major clinical therapeutic strategy for colorectal cancer (CRC) treatment especially advanced-stage cancer. Most chemotherapeutic agents are hydrophobic small molecules. The poor solubility and non-selective biodistribution of these drugs lead to limited accumulation in tumors, serious side effects and hence suboptimal therapeutic outcomes (Chang et al., 2020). An emerging strategy to improve the therapeutic efficacy of chemotherapy has been developed by nanotechnology and biomaterials (Luo et al., 2020; Wang et al., 2022). Highly hydrophobic drugs can be packed into highly water-soluble nanoscale delivery vehicles (10–100 nm diameter), and agents within this size range can easily permeate in solid tumors due to leaky tumor vasculature and poorly developed lymphatic drainage system in many solid tumors. However, the curative effect improvement remains poor (Izci et al., 2021).

The surface decoration of nanoparticles by tumor-targeting ligands could significantly enhance therapeutic efficacy and attenuate adverse effects via active targeting to tumor cells. Various targeting ligands including antibodies, peptides, aptamers, and small molecule inhibitors have been used to improve the targeting effect of nanodrugs (Wang et al., 2017; He et al., 2019; Zhu et al., 2022). Peptide-based therapeutic agents are gaining recognition as an important method for targeted drug delivery with improved antitumor efficacy and reduced side effects. For example, a peptide-drug conjugate DTX-P7, composed of docetaxel (DTX) and heptapeptide (P7, LPLTPLP), which specifically binds to cell surface Hsp90, is highly effective for non-small cell lung cancer (Jiang et al., 2022); CD44-specific A6 short peptide (KPSSPPEE) boosts target ability and anticancer efficacy of polymersomal epirubicin (EPI) to human multiple myeloma (MM) (Gu et al., 2019). The strategy usually exploits the biological activities or self-assembling potential of small targeting peptides to improve the anti-tumor efficacy of compounds.

Phage display technology is a robust approach for identifying cancer targeting peptides (Pappas et al., 2016; Deyle et al., 2017). At present, many tumor-specific binding peptides have been identified, such as RGD and NGR peptide (targeting to tumor vasculature), peptide CRRHWGFEFC (targeting to tumor microenvironment matrix metalloproteinases 9), YEQDPWGVKWWY peptide (targeting to tumor associated macrophages) (Ndinguri et al., 2012; Cieslewicz et al., 2013; Saw and Song, 2019). Using phage display technology, we previously identified a novel colorectal cancer specific binding peptide P-LPK (LPKTVSSDMSLN) (Hou et al., 2018). Here, we evaluate whether peptide P-LPK can be used as nanocarriers to delivery chemotherapeutic drugs for CRC targeted therapy. Paclitaxel (PTX) is a unique antimitotic agent with poor water solubility and indiscriminate distribution in normal tissues. Abraxane®, the first nanotechnology-based drug approved by FDA, is a non-covalently bound paclitaxel-human albumin nano-complex. Although Abraxane® can enhances intratumoral uptake, reduces the incidence of hypersensitivity and the dose of paclitaxel, they did not yield significant improvements in the clinical therapeutic index. On the other hand, Abraxane® cannot avoid the peripheral neuropathy induced by PTX. Similarly, the clinical outcomes of alternative PTX nano-formulations such as Lipusu®, Genexol-PM®, and Nanoxel® are not as satisfactory as expected. (Bernabeu et al., 2017; Sofias et al., 2017).

It was reported that paclitaxel were covalently linked to a brain peptide vector, Angiopep-2, to conduct a paclitaxel-Angiopep-2 conjugate named ANG1005. Preclinical and clinical evidences showed ANG1005 can cross the BBB, showing notable antitumor activity and well tolerated for patients with primary or metastatic brain tumors (Regina et al., 2008; Thomas et al., 2009; Kumthekar et al., 2020). In this study, we designed a novel self-assembled PTX-loaded nanoparticle (LPK-PTX NPs) based on the P-LPK peptide which selectively delivers PTX to the tumor site. First hydrophobic PTX was linked to peptide P-LPK by click reaction, and they further self-assemble into about 100-nm-sized spherical nanoparticles. The peptide P-LPK modification was shown to facilitate the uptake of the conjugated PTX in CRC cells, leading to higher cytotoxicity of PTX to CRC cells compared to normal cells. Notably, the *in vivo* data collected from CRC PDX model demonstrated that LPK-PTX NPs significantly enhanced antitumor activity and reduced systemic toxicity.

## Materials and methods

### Cell culture

The human CRC cancer cell lines HCT116 were purchased from the American Type Culture Collection. The human CRC cancer cell lines RKO and a normal human intestinal epithelial cell line NCM460 was obtained from the Chinese Academy of Sciences, Shanghai Branch. HCT116 were grown in McCoy’s 5A (Gibco) with 10% (v/v) foetal bovine serum. RKO, NCM460 cells were grown in RPMI 1640 (Gibco) supplemented with 10% (v/v) fetal bovine serum. All culture medium contained 100 U/mL penicillin, and 100 μg/mL streptomycin (HyClone, Utah, US) and cells were incubated at 37°C in a humidified 5% CO2 incubator.

### Synthesis of the peptide-PTX conjugate

The DBCO-NHS ester was dissolved in DMSO, and the P-LPK peptide or the Reverse peptide of P-LPK peptide (Named Rev, as a control peptide) solution was added drop wise under stirring. The mixture was stirred overnight to prepare a liquid phase to separate and purify the DBCO-peptide.

PTX (0.5 mmol, 426.5 mg), EDCI (0.6 mmol, 114.8 mg), and DMAP (0.6 mmol, 72.1 mg) were suspended in anhydrous dichloromethane (20 mL). The mixture was then cooled in an ice bath. A solution of 3-azidopropanoic acid (0.55 mmol, 66 mg) in anhydrous dichloromethane (5 mL) was added dropwise over a period of 20 min under stirring. The resulting solution was allowed to react at room temperature for 12 h. The reaction solvent was removed via rotary evaporators, and the residue was partitioned between distilled water (20 mL) and ethyl acetate (EA) (40 mL). The organic layer was separated and dried over anhydrous MgSO4, filtered, and concentrated via rotary evaporators to obtain the crude product, which was further purified via silica gel chromatography (hexane/EA = from 5:1 to 1:1, v:v) to afford 319 mg of the conjugate named PTX-N3, yield 67.5%.

Then DBCO-P-LPK peptide or DBCO-Rev peptide (100 mM) dissolved in 10 mL water and PTX-N3 (200 mmol) dissolved in 5 mL of DMSO and were mixed together and shaken at 50 C for 48 h. The two reaction mixture was then collected and purified by HPLC (MeOH/H2O), named LPK-PTX NPs and Rev-PTX NPs, respectively. The column used here was C18 reversed phase column and the eluent was MeOH and H2O. Elution condition was MeOH/H2O (v:v) from 5:95 to 80:20 (0.1% TFA).

Cy5.5 was labelled as follows. Firstly, Rev-PTX or LPK-PTX (2 mg) was added into 2 ml ultra-pure water. The solution was ultrasonic for 30 min. Then 1 µl Cy5.5 (5 mg/mL) was added into the solution and stirred overnight under room temperature. Eventually the precipitation was removed through high speed centrifugation. The obtained nanoparticles were stored at dark environment.

### Characterization of LPK-PTX nanoparticles

To determine nanoparticle size distribution, 1 ml sample was placed in a glass cuvette and dynamic lighting scattering (DLS) measurements was performed at room tempertature. Transmission electron microscopy (B-TEM, Tecnai G2 Spirit Biotwin) were also performed to investigate the morphology and size of nanoparticles.

For assessing nanoparticle stability, LPK-PTX NPs were placed in PBS, culture medium and culture medium (containing FBS) for 7 days. The size of nanoparticles was examined using DLS.

### PTX release experiment

The *in vitro* release behavior of LPK-PTX nanoparticles was investigated under PBS and culture medium at 37°C. A total of 3 ml of LPK-PTX nanoparticles was transferred into a dialysis bag (MWCO = 3,000 g/mol). The dialysis bag was put in the flask, immersed by 60 mL of pH 7.4 phosphate buffer (PBS) or culture medium, and stirred slightly at 37°C in the dark. Then, 2 mL of the external PBS buffer was replaced with 2 mL of fresh PBS immediately at predetermined time intervals (0, 2, 4, 6, 12, 24, 48 h), keeping the sinking condition. Here, the concentration of PTX in the external buffer was determined to analyze the amount of released PTX from nanoparticles.

HCT116 cells were seeded in culture dishes until the cell confluency reached the density of 85%. Then, LPK-PTX NPs were added to the culture medium at a concentration of 50 μM. After different time intervals of exposure, cells were collected and washed three times with ice-cold PBS. Cells were crashed via a cell scraper, and centrifugation and extraction by ethyl acetate were used to obtain the supernatant. The supernatant was analyzed by HPLC and calculated by analyzing standards of PTX.

### *In vitro* cellular uptake study

For confocal microscopy assay, HCT116 cells were seeded into single well confocal dish (NEST) at a density of 1 × 105 cells per well and cultured overnight. 20 μg/ml Cy5.5-Rev-PTX NPs or Cy5.5-LPK-PTX NPs were incubated for 0.5, 1, 2 and 4 h at 37°C. Then the cells were gently washed with PBS three times. Afterward, 20 mm Hoechst 33342 (ThermoFisher scientific) were added and incubated for 10 min for nuclei staining. For lysosome stain, lysotracker was added and incubated for 30 min. Finally, the cells were visualized through a Leica confocal microscope.

For flow cytometry analysis, HCT116 cells were seeded into six-well plates at a density of 1 × 105 cells per well. When cell confluence reached approximately 80%, the medium was removed and incubated with 20 μg/ml Cy5.5-peptide NPs for 1, 2, 4 h respectively. Then cells were washed by cold PBS thrice, and resuspended in 0.5 ml PBS before examined by the FACScan (Becton Dickinson, San Jose, CA, United States). At least 10,000 cells were collected and analyzed with the FACStation software program.

### *In vitro* cytotoxicity

*In vitro* cytotoxicity was determined by CCK8 assay (*n* = 5). NCM460 cells, HCT116 cells or RKO cells were seeded in 96-well plates at density of 3–5×103 cells per well and incubated at 37°C overnight. Then cells were treated with PTX, Rev-PTX NPs, or LPK-PTX NPs at various concentrations for 48 h. Afterward, the drug mediums were removed, and cells were rinsed gently with PBS for three times. Subsequently, 100 μl CCK8 solution was added in each well and incubated at 37°C for another 2 h. The absorbance of the solution was measured at 490 nm with a microplate spectrophotometer (BioTek, Winooski, United States). Cells without drug treatment were served as control.

### Cell apoptosis study

HCT116 cells were seeded in six-well plates at a density of 1 × 104 cells per well and incubated at 37°C until a confluence of 80%. The cells were treated with PTX, Rev-PTX NPs, LPK-PTX NPs at normalized PTX concentration of 5 μg/ml at 37°C for 48 h. Then the cells were trypsinzed, washed, and stained with Annexin V-Alexa Fluor488/PI according to manufacturer’s protocol (Life Technologies, United States). The cell apoptosis was monitored by flow cytometry (BD, CA, United States) and the apoptotic percentage of three independent experiments was analyzed.

### *In vivo* imaging

To establish human colon cancer xenografts, briefly, HCT116 cells were harvested using 0.05% trypsin solution and resuspended as single-cell suspensions in PBS at a concentration of 1 × 107 ml. 100 μl HCT116 suspension was injected subcutaneously at the dorsal side of nude mice.

The animal experiments were carried out in accordance with guidelines evaluated and approved ethics committee. The tumor volume was calculated as length*width2/2. After the tumor volume increased up to about 150 mm3, the animals were randomized into two groups and intravenously (i.v.) administered with 200 μl Cy5.5-Rev-PTX and Cy5.5-LPK-PTX at an equivalent dose of 20 μg/ml. The mice were anesthetized and visualized through an IVIS Spectrum system (Perkin Elmer, MA, United States) at Ex/Em 673/707 nm at 1,2, 4, 6, 12, 24 h post injection.

After *in vivo* imaging, the mice were sacrificed and the tumor, heart, liver, spleen, lung, and kidneys were harvested for further *ex vivo* fluorescence imaging using the same IVIS instrument.

To investigate the half-life of nanoparticles, Cy5.5-Rev-PTX NPs or Cy5.5-LPK-PTX NPs were intravenously injected into the tail vein of mice. Blood was withdrawn from the tail vein at predetermined time points (1, 2, 4, 8, 12, 24 h) and Cy5.5 fluorescence signal in blood was measured using a fluorescence spectrophotometer.

### *In vivo* therapeutic experiments

The therapeutic efficacy of was evaluated in PDX mice model. When the tumor volume reached about 100 mm3, mice were randomly assigned into four groups (six mice per group) and intravenously administered with DMSO, free PTX, Rev-PTX NPs and LPK-PTX NPs respectively at an equivalent dose of 0.8 mg/kg PTX every 3 days. Total mice body weights and tumor sizes were recorded every 3 days. At the end of the observation, tumors and major organs including heart, liver, spleen, lung, colon and kidney were isolated, deparaffinized and subjected to hematoxylin and eosin (H&E) and Ki-67 staining using standard protocols for histopathological analysis.

### TUNEL assay

The mice tumor tissue samples were fixed in 10% formalin, dehydrated by ethanol, and embedded in paraffin. DNA fragmentation of tissue sections was detected by a TUNEL kit (Roche, Germany) according to the manufacturer’s protocol.

### Statistical analysis

The statistical analyses were performed by using the GraphPad 7.0 statistical software. All the data were reported as mean ± standard deviation. Comparisons among multiple groups were assessed by one-way ANOVA, and *p < 0.05, **p < 0.01, ***p value <0.001, ****p < 0.0001 were considered significant. The statistical significance between all pairs of components was assessed using one-way analysis of variance followed by Tukey’s multiple comparisons post-hoc test.

## Results

### Characterization of the LPK-PTX NPs

**FIGURE 1:**
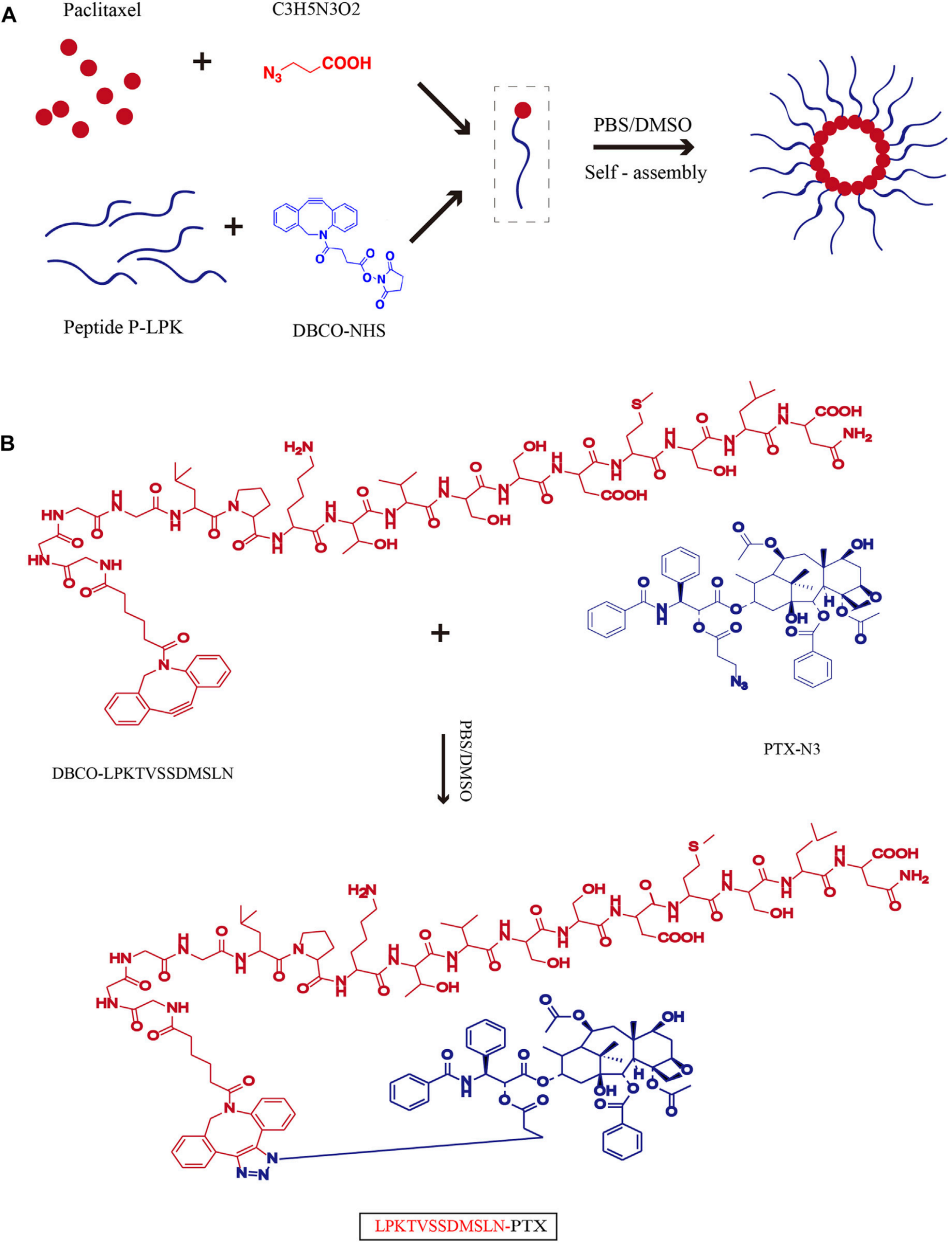
Schematic of the synthesis of LPK-PTX NPs. **(A)** Chemical Structure of LPK-PTX conjugate and schematic of the structure of LPK-PTX NPs. The peptide LPK was conjugated to PTX by a linker. Attachment of the hydrophobic drug PTX triggers self-assembly of the peptide LPK into spherical nanoparticles with a drug-rich (Red circles) core surrounded by a hydrophilic peptide corona (Blue chains). **(B)** PTX was first reacted with 3-azidopropanoic acid at the 2′-OH position of PTX to introduce an azide-carbonyl functional group. The peptide P-LPK was modified with DBCO. Then PTX-N3 and DBCO-P-LPK was conjugated by click reaction.

To activate PTX for conjugation, it was first reacted with 3-azidopropanoic acid at the 2′-OH position of PTX to introduce an azide-carbonyl functional group (Supplementary Figure S1, S2). Then the activated PTX were conjugated with DBCO modified peptide P-LPK through click reaction. Upon conjugation, the LPK-PTX conjugate spontaneously self-assembled into near-monodisperse spherical nanoparticles (Figure 1A). The reverse peptide of P-LPK (named Rev peptide) was conjugate with PTX as control, named Rev-PTX NPs.

**FIGURE 2:**
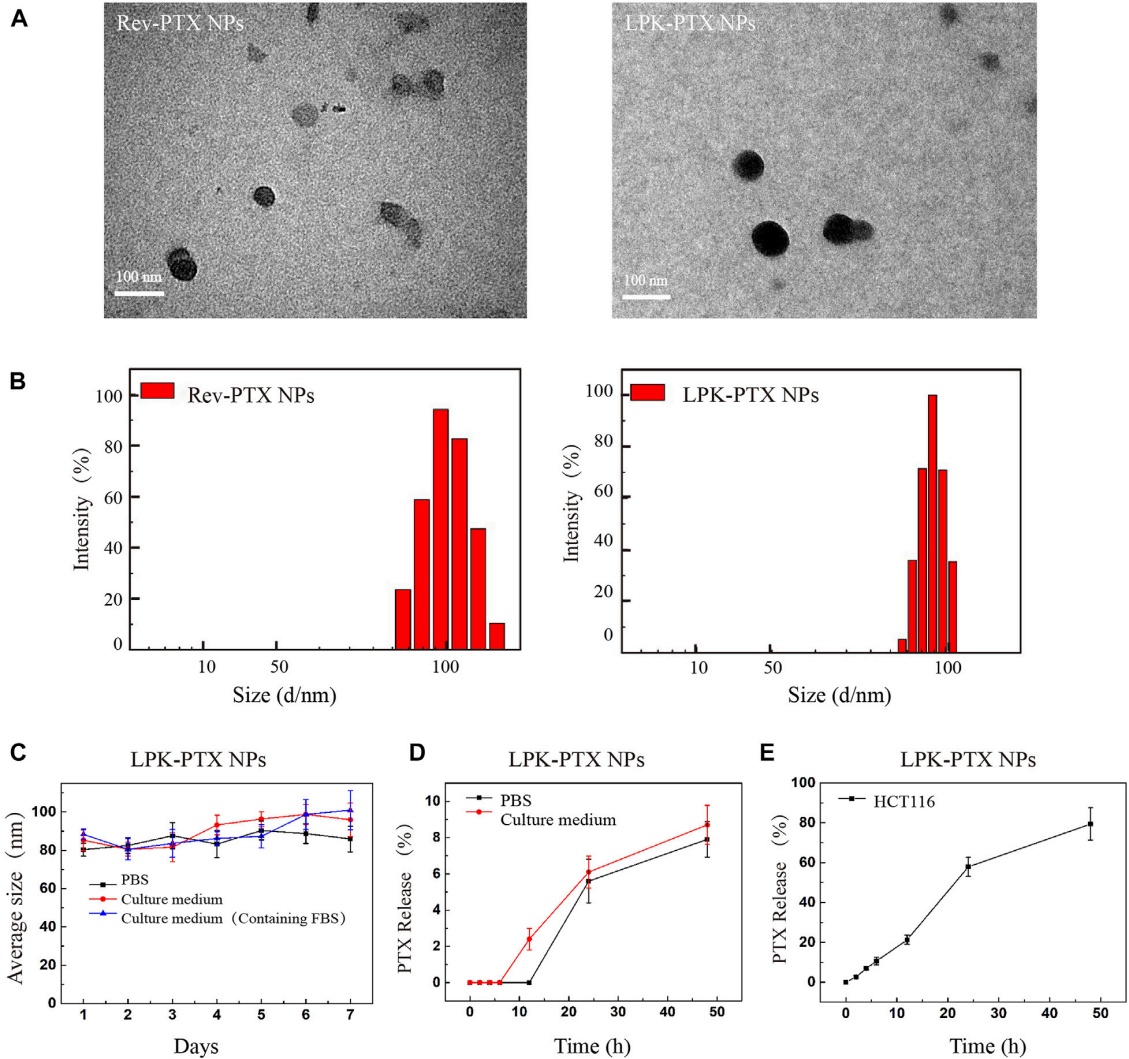
Characterizations of LPK-PTX NPs. **(A)** LPK-PTX NPs morphology were imaged via TEM. Black particles were Rev-PTX NPs or LPK-PTX NPs. **(B)** DLS studies showed particle size distribution of Rev-PTX NPs and LPK-PTX NPs (*n* = 3). **(C)** Size distribution of LPK-PTX NPs showing their stability in PBS, Culture medium and Culture medium (Containing FBS). The PTX release rate of LPK-PTX NPs in PBS, Culture medium **(D)** and HCT116 cells **(E)** were detected at different time points (0, 2, 4, 6, 12, 24, and 48 h).

The size and morphology of LPK-PTX NPs and Rev-PTX NPs were observed by transmission electron microscopy (TEM) for the direct visualization of self-assembled structures in a near-native state. TEM images showed the two NPs exhibited very similar size and shape, which were monodisperse nanoparticles and spherical (Figure 2A). The granule diameter was range 80–100 nm. The size distribution was further measured by dynamic light scattering (DLS). The results showed an average size of 100 and 82.1 nm for Rev-PTX NPs and LPK-PTX NPs, in deionized water (Figure 2B). The polydispersity index (PDI) of Rev-PTX NPs and LPK-PTX NPs were 0.27 and 0.12, respectively. Besides, there was no significant change in size in presence of PBS, culture medium, and culture medium (containing FBS) for 7 days, indicating that LPK-PTX NPs were stable in a neutral environment without manifesting aggregation or degradation (Figure 2C).

The stability of nanoparticles was further investigated under a simulated physiological condition at 37°C. The results showed that in PBS, PTX release was not detected before 12h, and the release rate gradually increased with time, reaching 7.9% at 48h; in culture medium (containing 10% FBS), the release rate of PTX gradually increased from 6 h and reached 8.7% at 48 h. Taken together, the PTX release rate in culture medium was higher than that in PBS after 6 h (Figure 2D). Next, we examined the release of PTX inside HCT116cells. As shown in Figure 2E, PTX was gradually released over time in HCT116 cells; more than 50% released at 24 h, and reached 79.4% at 48 h.

### The effect of P-LPK modification on cellular uptake

**FIGURE 3:**
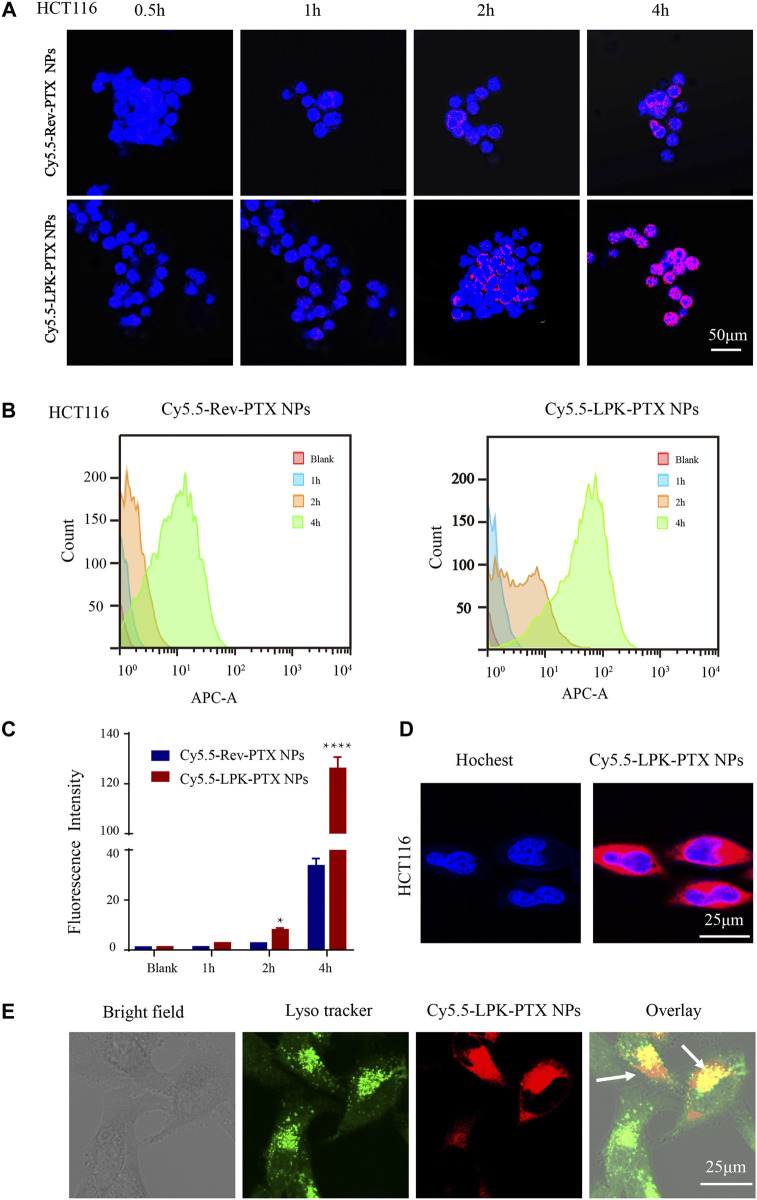
Cellular uptake of Cy5.5-LPK-PTX NPs *in vitro*. **(A)** Confocal laser scanning microscopy (CLSM) images of HCT116 cells incubated with 20 μg/ml Cy5.5-Rev-PTX NPs or Cy5.5-LPK-PTX NPs for 0.5, 1, 2, and 4 h, respectively. The fluorescence intensity of Cy5.5-LPK-PTX NPs was much stronger than Cy5.5-Rev-PTX NPs at various times. **(B, C)** Cellular uptake of Cy5.5-Rev-PTX NPs or Cy5.5-LPK-PTX NPs in HCT116 cells after incubation for 1, 2 or 4 h were detected by flow cytometry. And the fluorescence intensity in HCT116 cells was calculated (n = 3) (*p < 0.05, ****p < 0.0001). **(D, E)** Subcellular localization of LPK-PTX NPs. HCT116 cells were incubated with Cy5.5-LPK-PTX NPs for 4 h and treated with Hoechst for nuclear staining or Lysotracker for tracking lysomes. Then cells were observed by confocal microscopy. The overlay results indicated that LPK-PTX NPs was localized in the lysosome. Bar, 25 μM.

A near-infrared (NIR) fluorescent dye Cy5.5 NHS ester was used to label peptide. The uptake of Cy5.5-LPK-PTX NPs (Cy5.5-Rev-PTX NPs as control) was examined by confocal and flow cytometry. As shown in confocal laser scanning microscopy image, Cy5.5-LPK-PTX NPs displayed much higher fluorescence intensity in HCT116 cells, and the fluorescence intensity of Cy5.5-LPK-PTX NPs gradually increased from 0.5 to 4 h, suggesting that P-LPK facilitates the uptake of the conjugated PTX in HCT116 cells (Figure 3A). Interestingly, Cy5.5-Rev-PTX NPs showed a certain amount of uptake at 2 h after incubation, which could possibly because, there were non-specific interactions between peptide and cell surfaces.

Similar results were obtained from flow cytometry. As shown in Figures 3B,C, an increased fluorescence intensity of Cy5.5-LPK-PTX NPs was observed from 1 to 4 h. Compared to Cy5.5-Rev-PTX NPs, the fluorescence intensity of Cy5.5-LPK-PTX NPs was significantly higher at 2 and 4h, suggesting the uptake of Cy5.5-LPK-PTX NPs by HCT116 cells was much stronger than Cy5.5-Rev-PTX NPs (*p < 0.05, ****p < 0.0001).

Next, we observed the subcellular distribution of Cy5.5-LPK-PTX NPs. As shown in Figure 3D, after HCT116 cells incubated with Cy5.5-LPK-PTX NPs for 4 h, obvious intracellular red fluorescence were observed, suggesting that Cy5.5-LPK-PTX NPs were internalized into cells. Then green Lysotracker was utilized to further assess the cellular uptake. The red fluorescence of Cy5.5-LPK-PTX NPs was primarily found to be co-localized with the green signal of LysoTracker, indicating effective intracellular uptake and lysosomal entrapment of LPK-PTX NPs (Figure 3E).

### LPK-PTX NPs displaying an enhanced anti-tumor activity *in vitro*

**FIGURE 4:**
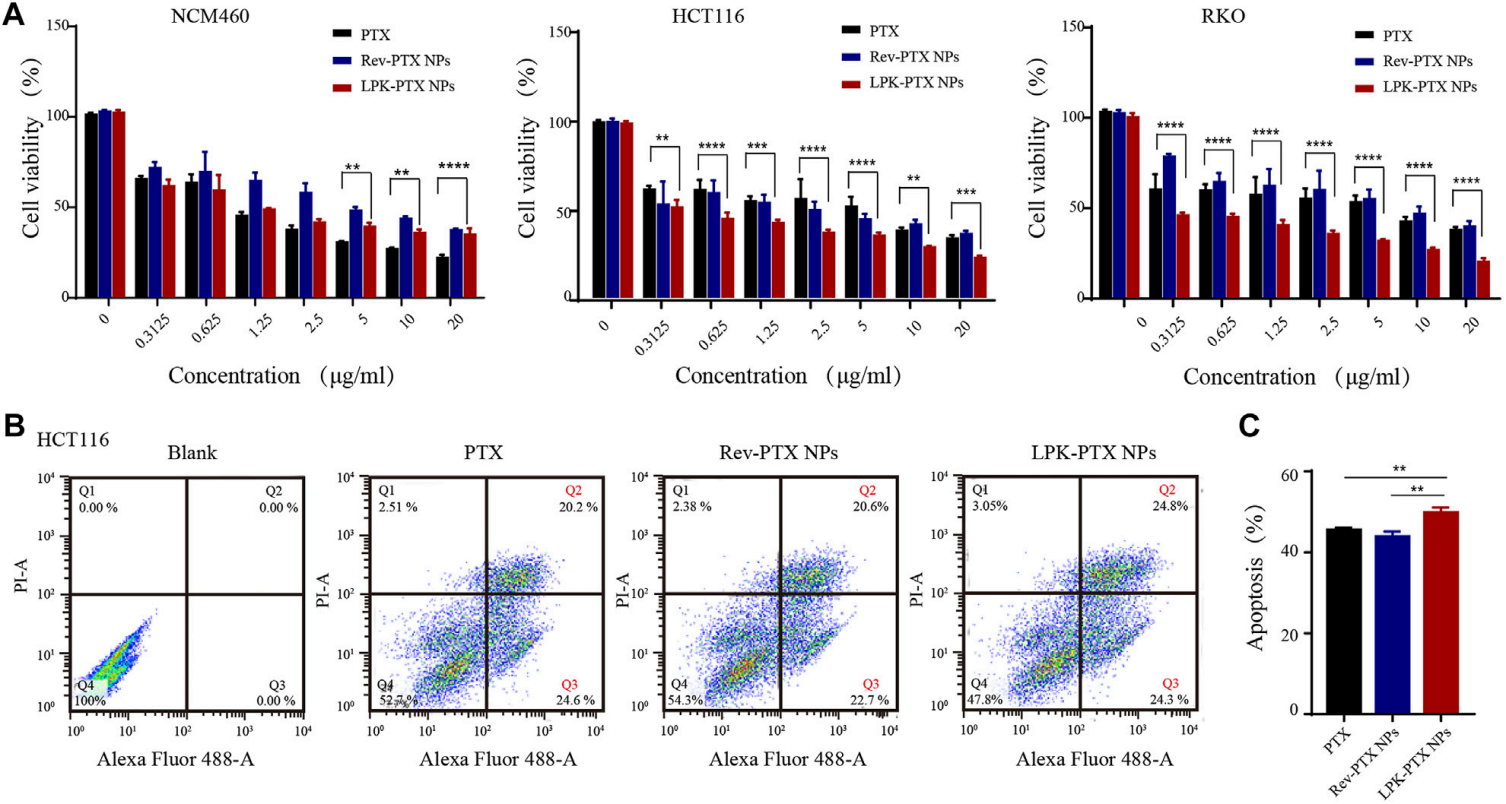
*In vitro* cytotoxicity of LPK-PTX NPs. **(A)** Cell viability of LPK-PTX NPs (PTX, Rev-PTX NPs as control groups) on normal cells NCM460 and colorectal cancer cells HCT116 and RKO after treated with various concentrations for 48 h. The concentration range of each compound was from 0.3125 μg/ml to 20 μg/ml. The data were presented as the means ± standard deviation. *n* = 5. *p < 0.05, **p < 0.01, ***p < 0.001, ****p < 0.0001. **(B)** Cellular apoptosis of HCT116 cells treated with PTX, Rev-PTX NPs and LPK-PTX NPs were investigated by flow cytometry. A representative results of early apoptosis (Q3: PI -, Annexin V+) and late apoptosis (Q2: PI +, Annexin V +) induced by PTX, Rev-PTX NPs, and LPK-PTX NPs were 44.8% (20.2% + 24.6%), 43.3% (20.6% + 22.7%), and 49.1% (24.8% + 24.3%), respectively. **(C)** The percentage early and late apoptosis was analyzed. (*n* = 3, **p < 0.01).

Cell viability and apoptosis assays were conducted to evaluate the antitumor efficacy of LPK-PTX NPs *in vitro*. After 48 h exposure, LPK-PTX NPs significantly inhibited the cell viability of HCT116 and RKO cells compared to free PTX (from 0.3125 μg/ml to 20 μg/ml) (Figure 4A). However, LPK-PTX NPs exhibited much lower cytotoxicity against human normal colon cells NCM460 cells from 5 μg/ml to 20 μg/ml, suggesting that LPK-PTX NPs is safer for normal cells (**p < 0.01, ***p < 0.001, ****p < 0.0001) (Figure 4A).

Cell apoptosis assays also reflected the superior tumor cytotoxicity of LPK-PTX NPs in HCT116 cells. A representative percentages of early apoptosis (Q3) and late apoptosis (Q2) induced by PTX, Rev-PTX NPs, and LPK-PTX NPs were respectively 44.8% (20.2% + 24.6%), 43.3% (20.6% + 22.7%), and 49.1% (24.8% + 24.3%) (Figure 4B). The apoptosis rate induced by LPK-PTX NPs was significantly higher than control groups, emphasizing the enhanced antitumor effect of LPK-PTX NPs (**p < 0.01) (Figure 4C).

### LPK-PTX NPs specific binding to human CRC cells *in vivo*

**FIGURE 5:**
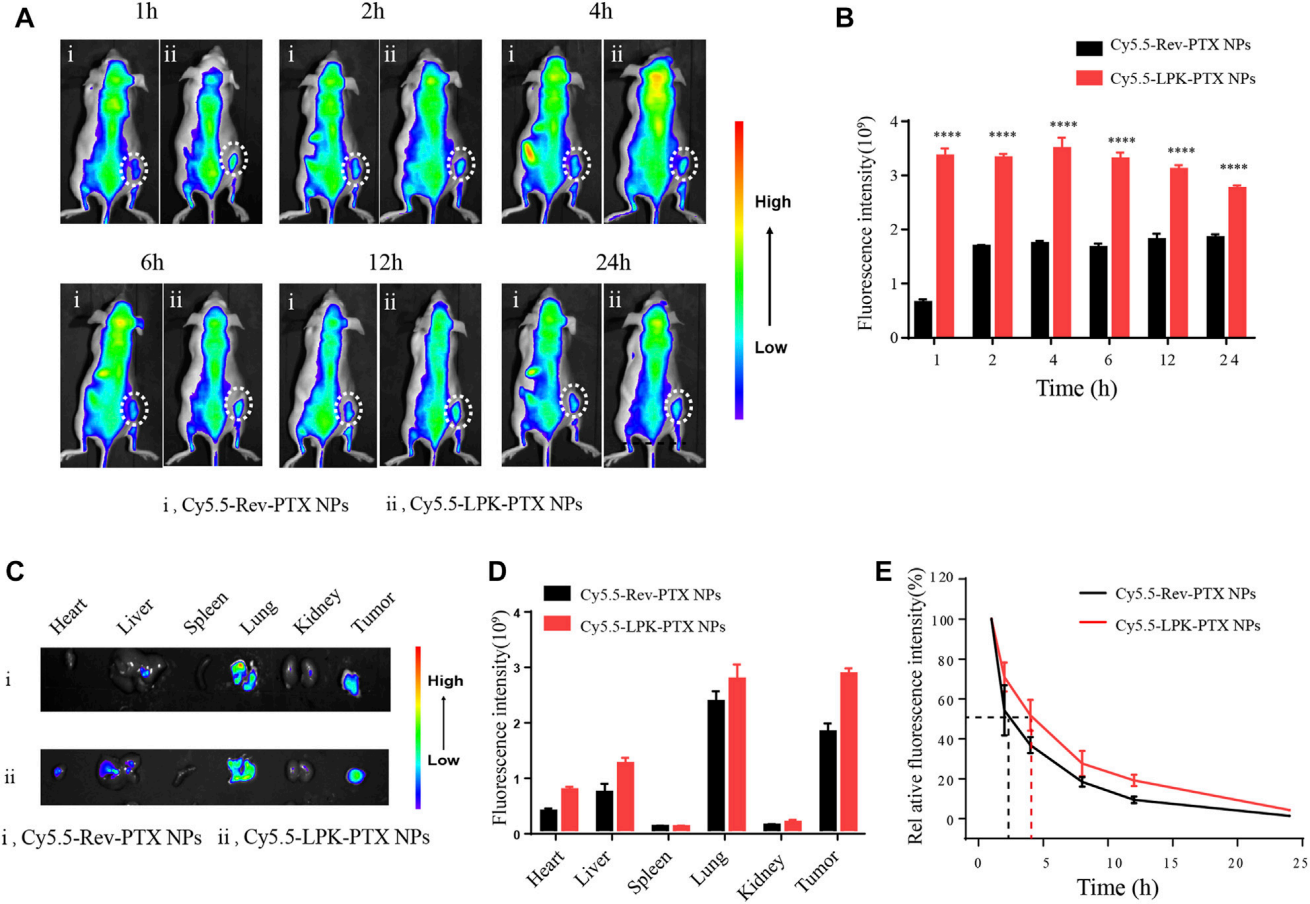
*In vivo* targeted tumor imaging of Cy5.5-LPK-PTX NPs. **(A)** The whole animal imaging of HCT116 tumor-bearing mice at 1, 2, 4, 6, 12, and 24 h after intravenously injection of 200 μl Cy5.5-Rev-PTX NPs or Cy5.5-LPK-PTX NPs (20 μg/ml). The circles indicate the locations of tumors in mice. **(B)** The fluorescence intensities of tumor tissues were quantified. ****p < 0.0001. **(C)** *Ex vivo* imaging of the tumors and other major organs (Heart, Liver, Spleen, Lung, Kidneys) from mice treated intravenously with Cy5.5-Rev-PTX NPs or Cy5.5-LPK-PTX NPs at 24 h. **(D)** The fluorescence intensities at the tumor sites and major organs were quantified. **(E)** The blood fluorescence intensity was detected at several time points post-administration.

The tumor-targeting property of LPK-PTX NPs was evaluated in a CRC xenografted mice model by *in vivo* imaging. As shown in Figures 5A,B, much higher fluorescence intensity was visualized at tumor sites in the Cy5.5-LPK-PTX NPs group all time points (1, 2, 4, 6, 12 and 24 h) after intravenous injection, revealing that Cy5.5-LPK-PTX NPs accumulated effectively in tumors. Moreover, the fluorescence at the tumor site can still be observed at 24 h post-injection, suggesting the high stability of Cy5.5-LPK-PTX NPs. To further evaluate the biodistribution, mice were sacrificed and the fluorescence intensity in major organs (Heart, Liver, Spleen, Lung, Kidney, Tumors) was quantitatively analyzed (Figures 5C,D). The results showed that tumors from the Cy5.5-LPK-PTX NPs group show much stronger fluorescence. Besides, the fluorescence intensity in some dissected organs (Heart, Liver, Lung) of Cy5.5-LPK-PTX NPs was much higher than that of Cy5.5-Rev-PTX NPs group (Figures 5C,D). This may be attributed to the different blood circulation time. As shown in Figure 5E, Cy5.5-LPK-PTX NPs had a half-life about 4h, whereas the half-life of Cy5.5-Rev-PTX NPs was about 2.5h, indicating a prolonged half-life of Cy5.5-LPK-PTX NPs in blood circulation.

### *In vivo* enhanced antitumor effect of LPK-PTX NPs

**FIGURE 6:**
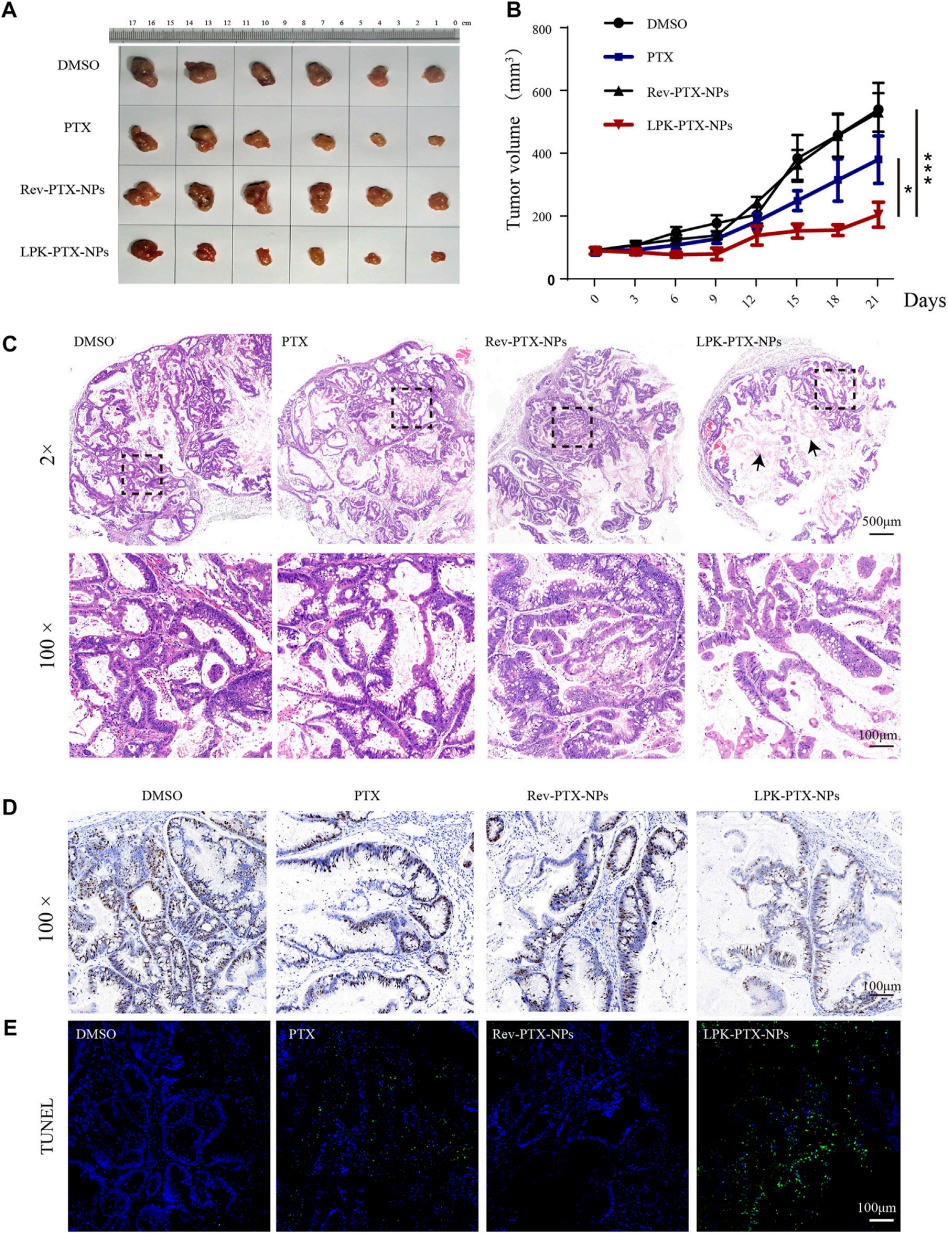
*In vivo* antitumor activity of LPK-PTX NPs. **(A)** The xenografted tumor after 21 days of different treatments from the groups indicated. **(B)** Analysis of tumor volume of different treatments via intravenous injection. The data were presented as the means ± standard deviation. *n* = 6, *p < 0.05. **(C)** H&E staining analysis of CRC sections from each group. Histologically, PDX tumor tissues preserved the heterogeneity of the original tumor. Tumor treated with the LPK-PTX NPs were substantially consists of connective tissues (Arrow indicated). Ki-67 **(D)** and TUNEL staining **(E)** in tumor tissue sections from different groups. Ki-67-positive staining was indicated by brown and nuclei were stained by blue. TUNEL-positive cells (Green) were significantly increased in LPK-PTX NPs group. Scale bar, 100 μM.

The *in vivo* antitumor effect of LPK-PTX NPs was evaluated in a patient-derived xenograft (PDX) model of CRC. Tumor-bearing animals were randomly assigned into four groups (Six mice per group) and intravenously administered with DMSO, free PTX, Rev-PTX NPs, and LPK-PTX NPs, respectively. The results showed that the average tumor volume of the LPK-PTX NPs treated group was significantly smaller than other groups (Figure 6A). And the tumor weight was significantly reduced in the P-LPK group compared to the controls (Supplementary Figure S3A). Especially, LPK-PTX NPs group showed a better inhibitory effect against PTX or Rev-PTX NPs group on 21 days after treatment (*p < 0.05, ***p < 0.001) (Figure 6B). Meanwhile, we observed tumor structure of four groups using H&E staining. The results showed that PDX tumor tissue maintained its original morphological features in each group, which is composed of cancer cells and connective tissue. Of note, larger proportion of connective tissue rather than CRC cells could be seen in the tumor sections of LPK-PTX NPs treated group, suggesting anti-tumor effect of LPK-PTX NPs is significantly enhanced (Figure 6C).

Then, Ki-67 staining, a commonly used cell proliferation marker, were performed to further investigate the tumor suppression efficiency of LPK-PTX NPs. We found numerous Ki-67-positive cells in tumor tissue from DMSO, PTX or Rev-PTX NPs treated mice, indicating active cell proliferation. In contrast, the LPK-PTX NPs treated group showed a lower Ki-67-positive cells, suggesting a higher inhibition effect on tumor growth (Figure 6D). TUNEL assay were also used to determine the cell apoptosis in tumor tissue. The results show that cell apoptosis (Green fluorescence) was remarkably enhanced in LPK-PTX NPs treated tumor tissue compared with other groups (Figure 6E).

### *In vivo* decreased toxicity of LPK-PTX NPs

**FIGURE 7:**
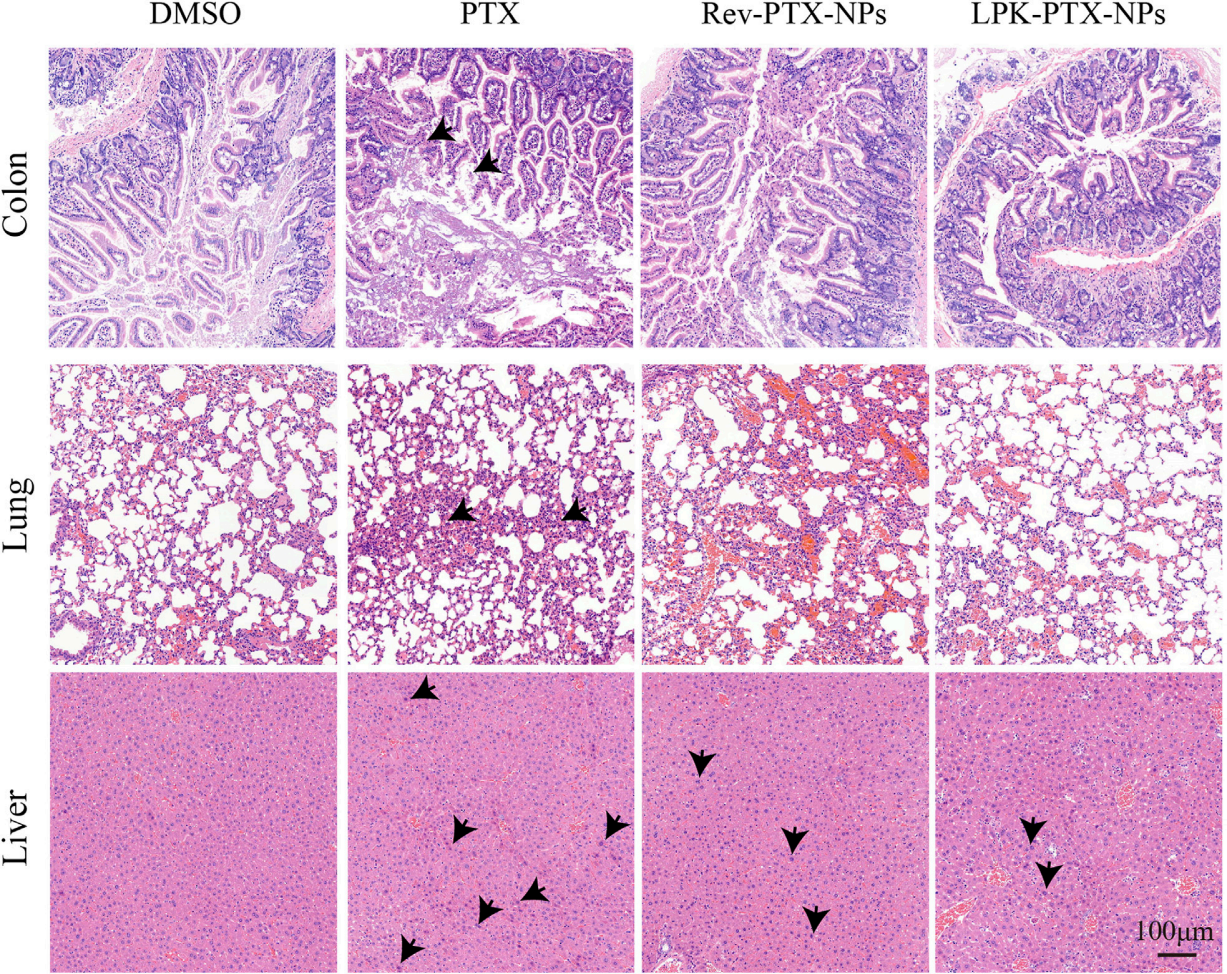
HE staining of colon, lung and liver in nude mice with different treatments. Colon crypt destruction was observed in PTX group but not in LPK-PTX NPs group. Apoptotic cells in liver and broken fibers in lungs were greatly reduced or absent in LPK-PTX NPs treated mice compared to PTX treated mice. The damages were indicated by arrows. Scale bar, 100 μm.

We further evaluate the systemic toxicity of LPK-PTX NPs in the PDX model. First, we measured the body weight of the treated mice every 3 days during the treatments. Importantly, negligible changes in body weight were observed during LPK-PTX NPs treatment, suggesting a slightly low potential of systemic toxicity (Supplementary Figure S3B). Next, PTX-related toxicities to major organs were evaluated by histology analysis with HE staining. As expected, serious damages were observed in the HE-stained sections of colon, lung and liver in the xenografted mice with PTX and Rev-PTX NPs treatment (Figure 7). Colons in LPK-PTX NPs group presented with normal crypt morphology, no signs of mucosal damage. However, severe crypt destruction occurred in mice treated with PTX. The apoptosis of liver cells and broken lung fibers were greatly reduced or absent in LPK-PTX NPs treated mice compared to PTX treated mice. Interestingly, there was no obvious damage in heart, spleen and kidneys from all the groups (Supplementary Figure S4).

## Discussion

Chemotherapy has shown remarkable therapeutic efficacy for CRC. However, adverse side effects reduce its effectiveness, leading treatment failure in many clinical indications. Increasing the efficacy and precision of chemotherapy entails molecular, that is, specific to cancer cells. To this end, we have previously screened a novel CRC specific peptide P-LPK using phage display technology. Herein, we successfully developed a novel self-assembled paclitaxel nanoparticle based on P-LPK for CRC targeted therapy.

Recently, peptides achieved resounding success in nano drug delivery (Spicer et al., 2018; Li et al., 2020). A selective MMP2 inhibitor (a novel peptide CCKIGLFRWR) was linked with doxorubicin (DOX) to produce an amphiphilic peptide-drug nanoparticle which can inhibit tumor metastasis and augment treatment efficacy (Qian et al., 2020). Fluorescent nanoparticles (f-PNPs) assembled by RGD cyclic peptides to deliver epirubicin (EPI) for esophageal cancer led to significantly reduced side effects and improved anti-tumor effect (Fan et al., 2018).

We previously identified a novel peptide P-LPK that was specifically binding to CRC cells. P-LPK peptide is extraordinarily efficient to synthesize and own a natural propensity to self-assemble into nearly monodisperse sub-100-nm nanoparticles in water upon conjugation of hydrophobic drugs. In this study, we first covalently linked peptide P-LPK with PTX to prepare a novel amphiphilic polymer (LPK-PTX conjugate). Then, the peptide-drug conjugate self-assembles in aqueous solution to form NPs.

A common fluorescent dye cyanine 5.5 (Cy5.5) NHS ester was labelled to LPK-PTX NPs for targeting capability assay. The cellular uptake of Cy5.5-LPK-PTX NPs was much higher than Cy5.5-Rev-PTX NPs in colorectal cancer cells at various times, suggesting that P-LPK played a crucial role in the entrance of the conjugated PTX into cancer cells. Subsequently *in vivo* imaging demonstrated that P-LPK facilitates selective targeting of LPK-PTX NPs to tumor tissues (Figure 5A). Moreover, LPK-PTX NPs can remain in the target site for a substantial amount of time (up to 24 h, the tumor site still showed obvious fluorescence) to avoid rapid clearance, and continue to exert its drug effects. Competition experiment further confirmed that P-LPK endows NPs with excellent targeting ability to specifically deliver PTX to CRC cells (Figure 5B). We believed that with development of super-resolution *in vivo* imaging, it may be possible to image the precise localization and even the subcellular localization of LPK-PTX NPs for monitoring drug delivery in live animals.

Herein, P-LPK and PTX are linked by click reaction, and then self-assemble into nanoparticles. We evaluated the antitumor efficacy of LPK-PTX NPs *in vitro*. Compared to PTX, LPK-PTX NPs showed superior cytotoxicity against CRC cells, indicating that after connecting with P-LPK, PTX maintains its anti-tumor activity and can be successfully released from LPK-PTX NPs. Meanwhile, LPK-PTX NPs exhibited less toxic than PTX in normal colon cells NCM460 (Figure 4A). In CRC PDX model, the LPK-PTX NPs treated mice showed smaller tumor size, less proliferation and more apoptosis in tumor tissue than control groups. Besides, it was notable that in tumor tissue of LPK-PTX NPs treated mice, the proportion of CRC cells was significantly reduced, which was mainly composed of tumor connective tissue (Figure 6C). This phenomenon further revealed that LPK-PTX NPs showed enhanced cytotoxicity to CRC *in vivo*. More importantly, compared with the control groups, no obvious tissue damage or any other side effects were observed in spleen, lung, liver, heart, intestines and kidney of LPK-PTX NPs treated mice. Furthermore, the toxicity of LPK-PTX NPs to liver and intestines is significantly lower than that of PTX, indicating that P-LPK effectively reduces the side effects of PTX and is considered to be safe for *in vivo* applications.

Peptide with a flexible structure and low molecule weight can be easily modified to improve the drug loading and pharmacokinetics. Besides, peptides are easier to synthesize for precise large-scale production. In this study, P-LPK, a specific CRC binding peptide, was evaluated as potential delivery systems of the anti-cancer agents PTX, providing a crucial strategy for tumor therapy. The specific peptide P-LPK could revive the application of PTX as a powerful chemotherapeutic agent by reducing its system toxicities. We believe that this specific feature makes P-LPK attractive for the delivery of hydrophobic chemotherapeutics that are limited in clinic because of their unfavorable physicochemical properties and poor bioavailability. At present, tremendous progress has been made in peptide delivery, but further development is necessary to translate basic research into preclinical or clinical investigation and finally enter the market. Multiple challenges such as stability, biocompatibility, targeting efficiency, and immunogenicity still need to be properly addressed.

## Conclusions

In summary, a novel peptide P-LPK was applied as a nanocarrier to load and deliver PTX specifically targeting CRC cells. LPK-PTX NPs showed enhanced efficacy while simultaneously reducing side effects. Importantly, since the conjugation interactions between P-LPK and drugs are not limited to PTX, P-LPK may be employed in principle for loading and tracking many hydrophobic drugs for enhanced anti-tumor efficiency and reduced the side effects.

## Data Availability

The original contributions presented in the study are included in the article/Supplementary Material, further inquiries can be directed to the corresponding author.
