# Supplementary material for: Self-assembled peptide-paclitaxel nanoparticles for enhancing therapeutic efficacy in colorectal cancer
Source: Front Bioeng Biotechnol. 2022 Sep 28;10:938662. doi: 10.3389/fbioe.2022.938662 (PMC9554092; doi:10.3389/fbioe.2022.938662)
Supplement: Supplementary file 1 [file DataSheet1.pdf]

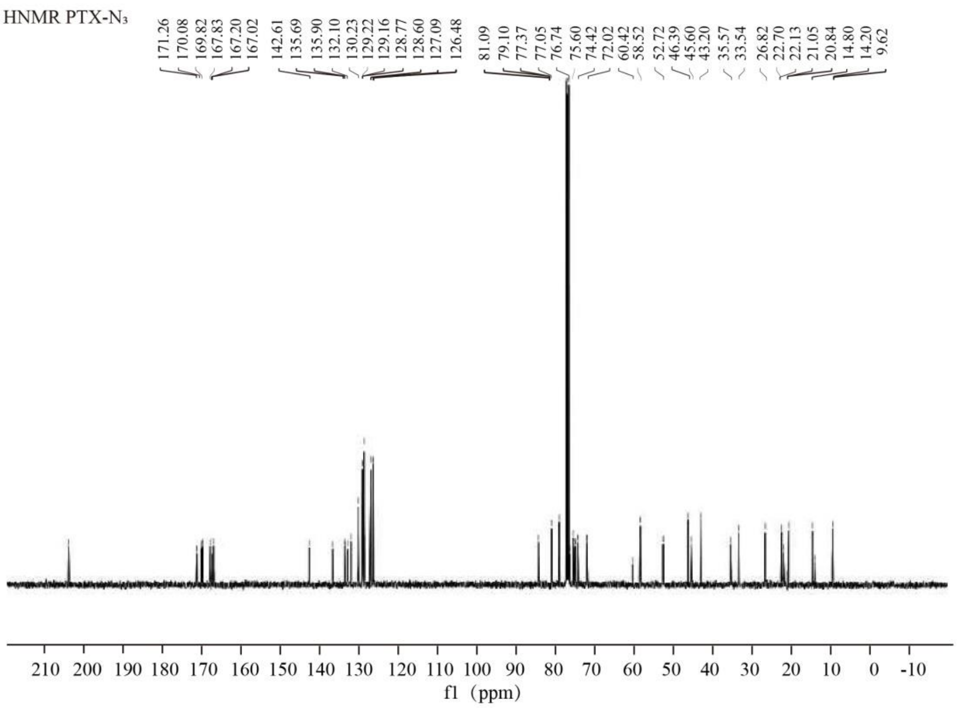

Supplementary Figure 1 ^{13}CNMR spectrum of PTX-N_3

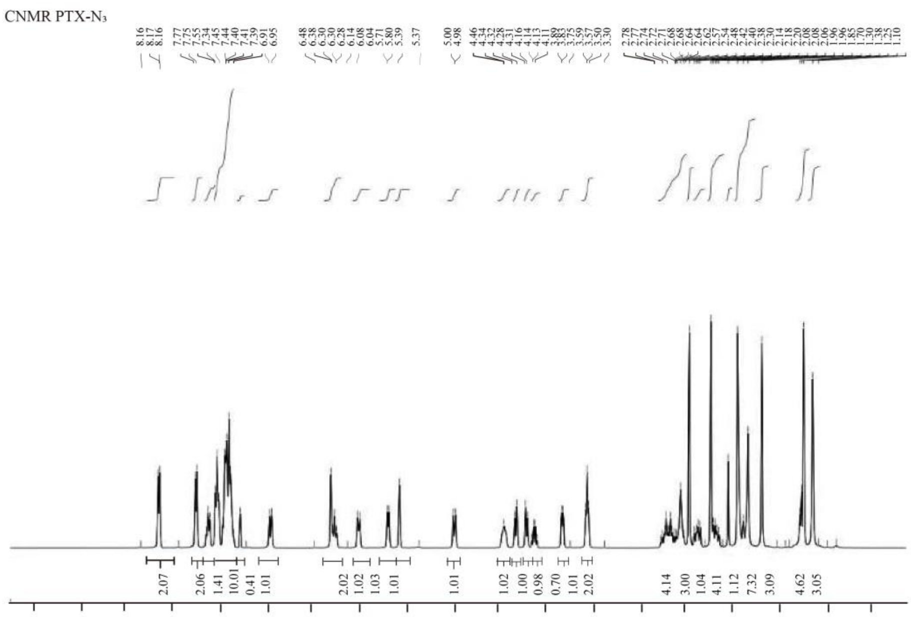

Supplementary Figure2 ^{1}H NMR spectrum of PTX-N_3

^{1}H NMR (400MHz, DMSO) δ ^{1}H NMR (400 MHz, DMSO) δ 8.16 (d, J = 8.0 Hz, 2H), 7.76 (d, J = 8.0 Hz, 2H), 7.65-7.61 (m, 1H), 7.55-7.36 (m, 11H), 6.96 (d, J = 8.0 Hz, 1H), 6.49-6.25 (m, 2H), 6.02 (d, J = 8.0 Hz, 1H), 5.7 (d, J = 8.0 Hz, 1H), 5.58(s, 1H), 4.99 (d, J = 8.0 Hz, 1H), 4.46-4.32 (m, 1H), 4.22 (d, J = 8.0 Hz, 1H), 4.15 (d, J = 8.0 Hz, 1H), 4.14-4.11 (m, 1H), 3.84-3.83 (m, 1H), 3.59-3.56 (m, 2H), 2.78-2.64 (m, 4H),

2.54 (s, 3H), 2.48-2.42 (m, 1H), 2.40 (s, 3H), 2.38-2.36 (m, 1H), 2.24 (s, 1H), 1.96 (s, 3H), 1.94-1.90 (m, 4H), 1.70 (s, 3H), 1.29-1.25 (m, 5H), 1.09 (s, 3H). ^{13}C NMR (100 MHz, $CDCl_3$, 298K) δ (ppm): 167.0, 133.7, 132.1, 130.2, 129.2, 129.1, 128.7, 127.1, 126.4, 84.5, 81.0, 79.1, 77.3, 77.0, 76.7, 75.6, 75.1, 74.4, 72.1, 72.0, 58.5, 52.7, 46.4, 45.6, 43.2, 35.6, 26.8, 22.7, 20.8, 12.8, 9.6.

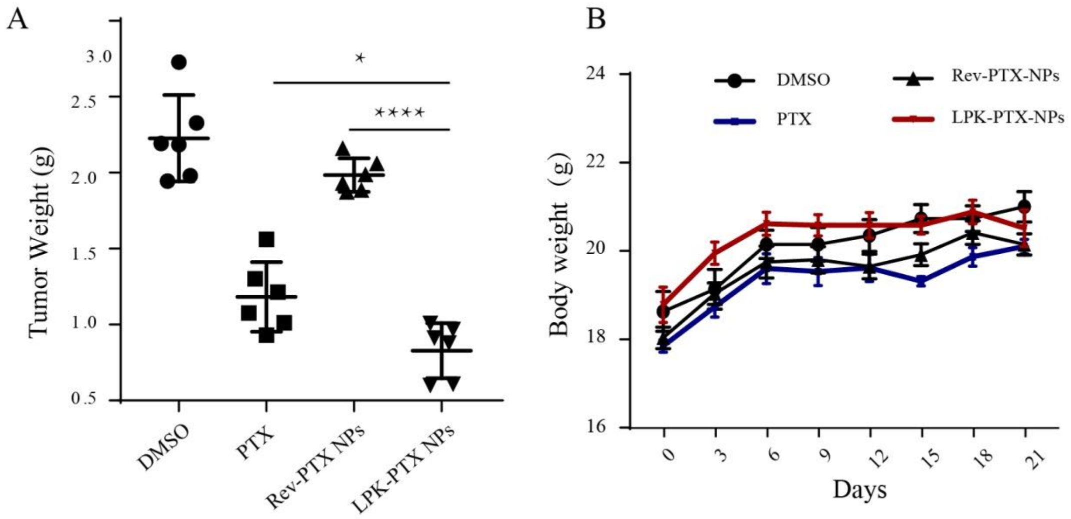

Supplementary Figure 3 Tumor weight and Body weight of mice with different treatments.

(A) The tumor weight was measured in each group. The tumor weight significantly reduced in the P-LPK group compared to the controls (*$p < 0.05$, ****$p < 0.0001$). (B) Body weight changes with different treatments. Body weight was normalized to each body weight before treatment.

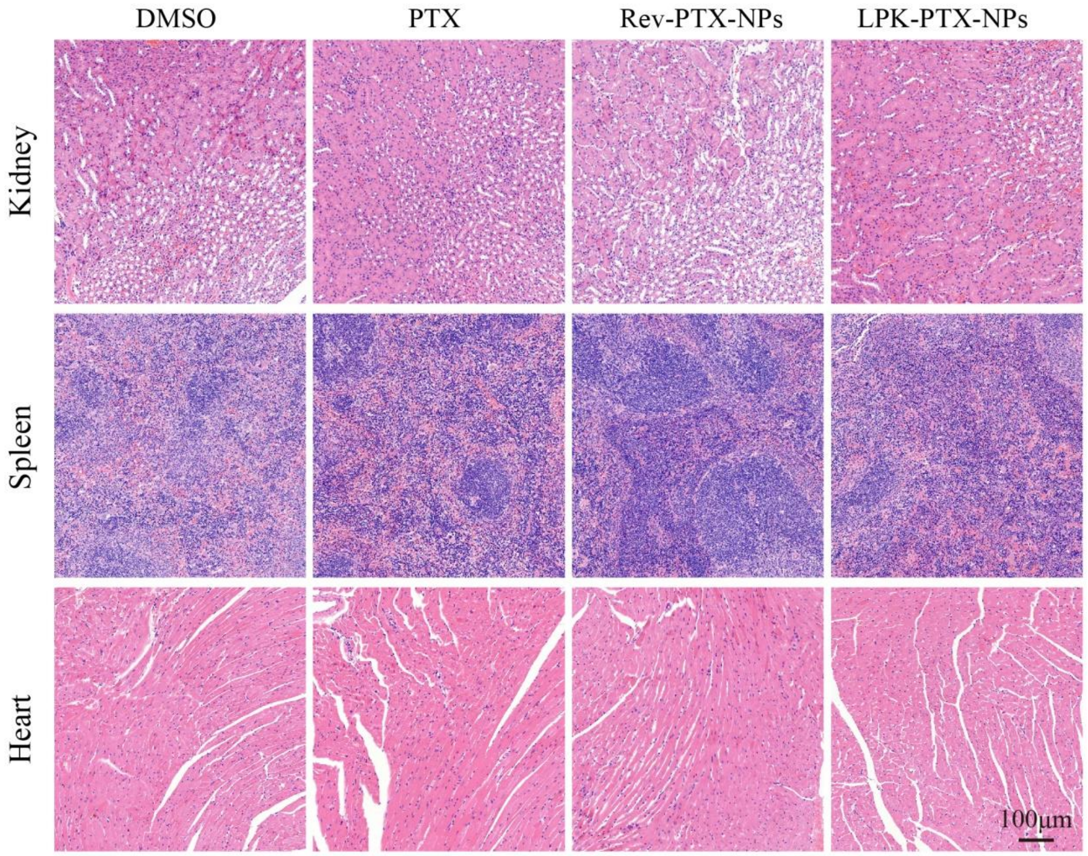

Supplementary Figure 4 HE staining of heart, spleen and kidneys in nude mice with different treatments. No obvious damage in heart, spleen and kidneys from all the groups. Scale bar, 100μm.
